# Treatments and Predictors of Mortality for Carbapenem-Resistant Gram-Negative Bacilli Infections in Malaysia: A Retrospective Cohort Study

**DOI:** 10.3390/tropicalmed7120415

**Published:** 2022-12-02

**Authors:** Usman Abubakar, Amni Izzati Zulkarnain, Jesús Rodríguez-Baño, Norhidayah Kamarudin, Mahmoud E. Elrggal, Mohamed Hassan Elnaem, Sabariah Noor Harun

**Affiliations:** 1 Discipline of Clinical Pharmacy, School of Pharmaceutical Sciences, Universiti Sains Malaysia, George Town 11800, Penang, Malaysia; 2 Faculty of Pharmacy, International Islamic University Malaysia, Kuantan 25200, Pahang, Malaysia; 3 Infectious Diseases and Microbiology Unit, Hospital Universitario Virgen Macarena, Department of Medicine, Biomedicines Institute of Seville, University of Seville, 41009 Seville, Spain; 4 Department of Pathology and Laboratory Medicine, Kulliyah of Medicine, International Islamic University Malaysia, Kuantan 25200, Pahang, Malaysia; 5 Department of Clinical Pharmacy, College of Pharmacy, Umm Al-Qura University, Makkah 21955, Saudi Arabia; 6 Quality Use of Medicines Research Group, Department of Pharmacy Practice, Faculty of Pharmacy, International Islamic University Malaysia, Kuantan 25200, Pahang, Malaysia

**Keywords:** carbapenem-resistant gram negative bacilli, carbapenem-resistant *Acinetobacter baumannii*, carbapenem-resistant *Enterobacteriaceae*, carbapenem-resistant *Pseudomonas aeruginosa*, in-hospital mortality, risk factors

## Abstract

This study evaluated the treatments, mortality rate and patient-related factors associated with mortality. This is a retrospective study involving hospitalised patients with infections caused by carbapenem-resistant Gram-negative bacilli (CR-GNB) in a tertiary hospital in Malaysia from January 2018 to June 2020. A clinical pharmacist reviewed patients’ electronic records and collected the data according to a pre-designed form. Data were analysed using both descriptive and inferential tests. The study included 145 patients with CR-GNB infections including 77, 40 and 28 *Acinetobacter baumannii*, enterobacteriaceae and *Pseudomonas aeruginosa*, respectively. The mean age was 57.9 ± 15.8 years. Pneumonia (40.7%) and bacteremia (25.5%) were the most common infections. Meropenem (24.7%) and piperacillin-tazobactam (20.4%) were the most commonly used empiric antibiotics while colistin (63.3%) and amikacin (8.3%) were the most common definitive antibiotics. The mean duration before active antibiotics was 4.6 ± 3.3 days. Overall, the in-hospital mortality rate was 41.4%. Multivariate logistic regression analysis showed that intensive care unit (ICU) admission (adjusted odds ratio (AOR): 5.201; 95% confidence interval (CI): 1.603–16.872; *p* = 0.006), sepsis/septic shock (AOR: 3.430; 95% CI: 1.021–11.522; *p* = 0.049) and elevated serum creatinine (AOR: 2.752; 95% CI: 1.005–7.536; *p* = 0.049) were independently associated with mortality. The mortality rate among patients with CR-GNB infection is high. A high rate of inappropriate antibiotic use was observed, including combination antibiotic therapy and delays in starting active antibiotics. Mortality was significantly associated with ICU admission, sepsis/septic shock and elevated serum creatinine.

## 1. Introduction

Carbapenems are one of the last-lines of antibiotics used for the treatment of serious infections caused by multidrug resistant Gram-negative pathogens, including the extended-spectrum beta-lactamase inhibitor positive pathogens [1,2]. An increase in the use of carbapenems has led to the emergence and spread of carbapenem-resistant pathogens. Carbapenem-resistant Gram-negative bacilli (CR-GNB) infections have become common in the last decade and they are rapidly spreading in healthcare settings posing serious threats to public health worldwide [3]. CR-GNB cause a wide range of infections, including bacteremia, urinary tract infections, pneumonia and intra-abdominal infections [4]. Carbapenem-resistant Enterobacteriaceae (CRE), carbapenem-resistant *Acinetobacter baumannii* (CRAB) and carbapenem-resistant *Pseudomonas aeruginosa* (CRPA) are ranked at the top of the global antibiotic-resistant bacteria priority list [5]. In addition, the Centers for Disease Control and Prevention (CDC) has classified CRE and CRAB along with multidrug-resistant *P. aeruginosa* as urgent and serious threats, respectively [6].

Although, the mechanism of resistance and the risk factors for CRAB, CRPA and CRE infections vary, there are some common risk factors associated with infections caused by these pathogens including intensive care unit (ICU) admission, length of hospitalisation and previous exposure to carbapenems [7,8,9,10,11,12]. The acquisition of carbapenem-hydrolysing β-lactamases or carbapenemases is the most common and clinically important mechanism underlying carbapenem resistance, which causes resistance to carbapenems as well as other β-lactam antibiotics [13]. The therapeutic options for the treatment of carbapenem-resistant infections are extremely limited. Carbapenem-resistant infections are associated with significant morbidity and mortality [4]. Previous studies have shown that mortality rate ranges from 48% to 57.4% for CRE [14,15], 52.7% for CRAB [7,16] and 8% to 18.4% for CRPA [17]. In addition, carbapenem-resistant infections are associated with longer hospitalisation [18], while the total excess cost of treatment is estimated to be USD 3966 per patient [19].

In Malaysia, inappropriate use of antibiotics among patients has been reported [20,21,22]. About 50% of antibiotic prescriptions among inpatients are non-compliant with guidelines [20]. This contributes to the emergence and spread of antimicrobial resistance. In addition, evidence has shown that carbapenems are commonly used for the treatment of extended-spectrum beta-lactamase producing *Enterobacteriaceae* [23]. The prevalence of carbapenem-resistant *Enterobactaeriaceae* ranged between 0.3% and 5.7% [24,25]. This highlights an important antimicrobial stewardship opportunity for hospital pharmacists to promote rational use of antibiotics and prevent antimicrobial resistance. Understanding the treatment outcomes and the factors associated with treatment failure and mortality is important to design effective interventions to improve treatment outcomes among hospitalised patients in Malaysia. Therefore, the objective of this study is to describe the treatments, mortality rate and factors associated with mortality among hospitalised patients with CR-GNB infections

## 2. Materials and Methods

### 2.1. Study Design and Inclusion Criteria

A retrospective cohort study including all hospitalised patients with infections due to carbapenem-resistant *Enterobacteriaceae* (CRE), carbapenem-resistant *Acinetobacter baumannii* (CRAB) and carbapenem-resistant *Pseudomonas aeruginosa* (CRPA) from January 2018 to June 2020 was conducted in a 350-bed tertiary hospital providing medical, surgical, intensive care, pharmacy, laboratory, nursing and orthopaedic care, located in the eastern coast of Malaysia. A list of all patients with infections due to CRE, CRAB and CRPA was obtained from the medical microbiology unit of the hospital. Patients with CRE, CRAB and CRPA colonisation and those with positive CRE, CRAB and CRPA culture without hospitalisation were also excluded.

### 2.2. Data Collection, Variables and Definitions

The data were collected by a clinical pharmacist who was experienced in antimicrobial stewardship and infectious diseases. The electronic medical records of the patients were reviewed to collect the following information: age, gender, race, ward of admission, type of infection (hospital/community-acquired), dates of admission, onset of symptoms and discharge or death, recent hospitalisations, recent surgery, presence of comorbidities, source/type of infection [26] and presence of sepsis or shock [27]. Other information collected was microbiological data such as the specimen collected, pathogen isolated and its susceptibility pattern; laboratory parameters; treatment-related data such as empiric and definitive antibiotics; and outcome data (microbiological cure, discharge/transfer or death). The main outcome variable was in-hospital all-cause mortality. A patient was considered immunosuppressed by fulfilling any of the following criteria: presence of neutropenia, leukaemia, lymphoma and HIV infection with less than 200 CD4/mL, solid organ or hematopoietic stem cell transplantation, cytotoxic chemotherapy, steroids (>15 mg of prednisone daily for > 2 weeks) [28]. Antimicrobial susceptibility was performed by automated system (Vitek 2) and interpreted using Clinical and Laboratory Standards Institute (CLSI) recommendations during the period under review. The study protocol was approved by the International Islamic University Malaysia Research Ethics Committee (IIUM/504/14/11/2/IREC 2020-099). Informed consent was waived due to the retrospective nature of the study. The study was conducted in accordance with the Declaration of Helsinki guidelines. The data were de-identified before data analysis

### 2.3. Data Analysis

Data were analysed using Statistical Package for Social Sciences (SPSS) software version 22. Data were de-identified before analysis. Categorical data were presented as frequency and percentage, while continuous data were presented as mean and standard deviation. The association between patients’ demographics, clinical, laboratory and treatment characteristics and in-hospital mortality was determined with univariate analysis using Chi-squared or Fisher test for categorical variables, and Student’s T-test or Mann–Whitney U test for continuous variables, as appropriate. Variables with a univariate *p*-value < 0.1 were considered for inclusion in multivariate logistic regression analysis; the variables were selected using a stepwise backward process. A value of *p* < 0.05 was considered statistically significant.

## 3. Results

### 3.1. Demographic and Clinical Characteristics of the Patients

A total of 145 patients were included in this analysis with a mean age of 57.9 ± 15.8 years and a male (68.3%) and Malay race (88.3%) preponderance (67.5%). More than 50% of the patients had intensive care unit admission. A history of carbapenem use in the preceding 30 days was noted in 40% of the cases. Pneumonia (40.7%) and bacteremia (25.5%) were the most common CRGNB infections. Sepsis/septic shock was reported in 64.1% of the patients, and 73.8% of the infections were hospital-acquired in nature. Table 1 summarises the characteristics of the patients included in the study.

### 3.2. Patients’ Laboratory Data and Vital Signs on the Day of Infection

Of the 145 patients, 11.0% were unconscious on the first day of the infection, and 53.8% were intubated. More than two-thirds had elevated total white blood cell count (71.7%) and low haemoglobin count (80.0%). In addition, more than 50% had elevated serum creatinine, urea and liver enzymes. The mean lowest systolic and diastolic blood pressure was 108 ± 22.1 and 58.6 ± 12.6, respectively (Table 1).

### 3.3. Microbiological Characteristics of the Infections

*A. baumannii* was the most common CR-GNB infection and represented 53.1% (*n* = 77), followed by *K. pneumoniae* (22.8%; *n* = 33) and *P. aeruginosa* (19.3%; *n* = 28). Twenty-one (14.5%) of the cases had concurrent fungal infections, of which 12 (57.1%) were secondary fungal infections (occurred after diagnosis of CR-NGB infection). One in five CR-GNB infections was polymicrobial in nature with *Enterobacterales*, *S. aureus* and *Candida* spp. being the most common pathogens involved. Table S1 in the supplementary material summarises the microbiology characteristics of the infections. Overall, there was more than 85% resistance of CRGNB pathogens to penicillins, penicillin plus beta-lactamase inhibitor combinations and second, third and fourth generation cephalosporins. Susceptibility to cotrimoxazole, gentamicin, amikacin and colistin was 17.6%, 23.7%, 26.2% and 100%, respectively. The sensitivity to gentamicin was significantly higher among CRPA (42.3%) and CRE (40.0%) isolates compared to CRAB (10.4%) isolates, *p* < 0.001. In addition, CRE (55.0%) and CRPA (42.9%) isolates were more susceptible to amikacin compared to CRAB (11.3%) isolates, *p* < 0.001. Conversely, susceptibility to cotrimoxazole was significantly higher among CRAB isolates (32.1%) compared to CRE (7.5%) and CRPA (0.0%) isolates, *p* = 0.042. Table S2 illustrates the antibiotic susceptibility patterns of CRGNB pathogens.

### 3.4. Empirical and Definitive Antibiotic Therapy for Carbapenem-Resistant Infections

A wide range of antibiotics was used for empirical therapy among the patients, with meropenem (24.7%) and piperacillin-tazobactam (20.4%) being the most commonly used. A combination of empirical antibiotics was used in 37.9% of the infection. Effective empirical antibiotics were observed in 11% of the infections. Colistin (63.3%) was the most commonly used definitive antibiotic among the patients, followed by amikacin (8.3%). About 41% received combination definitive antibiotic therapy including colistin-based (75.8%) and non- colistin-based (24.2%) combinations. The mean duration before effective antibiotics (the duration between onset of infection and the use of active antibiotics) was 4.6 days. Table 2 shows the empirical and definitive antibiotics used to treat CRGNB infections.

### 3.5. Treatment Outcomes and Univariate Regression Analysis for Factors Associated with in-Hospital Mortality

Overall, in-hospital and 14-day mortality rates were 41.4% and 23.4%, respectively. Univariate logistic regression analysis showed that ICU admission (odds ratio (OR): 10.766; 95% confidence interval (CI): 4.537–25.544; *p* < 0.001), female gender (OR: 2.842; 95% CI: 1.383–5.842; *p* = 0.004), sepsis/septic shock (OR: 5.802; 95% CI: 2.539–13.257; *p* < 0.001), elevated creatinine (OR: 2.881; 95% CI: 1.411–5.881; *p* = 0.004) and thrombocytopenia (OR: 2.302; 95% CI: 1.025–5.170; *p* = 0.043) were significantly associated with in-hospital mortality (Table 3).

### 3.6. Multivariate Regression Analysis for the Predictors of Mortality

Multivariate logistic regression analysis revealed ICU admission (AOR: 5.201; 95% CI: 1.603–16.872; *p* = 0.006), sepsis/septic shock (AOR: 3.430; 95% CI: 1.021–11.522; *p* = 0.049) and elevated serum creatinine (AOR: 2.752; 95% CI: 1.005–7.536; *p* = 0.049) were independently associated with in-hospital mortality among the patients. Table 4 describes the multivariate logistic regression analysis for factors associated with in-hospital mortality.

## 4. Discussion

The current study showed that CRAB is the most common CR-GNB infection and that in-hospital mortality among patients with CR-GNB infections was high and associated with ICU admission, sepsis/septic shock and elevated serum creatinine. Colistin-based combination was the most commonly used definitive combination therapy. The study found that the mean age of the patients was about 58 years with a male preponderance. These results are consistent with previous studies for age [29,30] and gender [29,31], respectively. Older adults have a higher risk of CR-GNB infections due to multiple comorbidities, frequent hospitalisation and frequent exposure to antibiotics [15]. A previous study reported that prior use of carbapenem is an independent risk factor for CR-GNB infection [18]. Similarly, the current study found that more than one-thirds of the patients had used carbapenem antibiotics within 30 days prior to the isolation of a CR-GNB pathogen. The mechanism of carbapenem resistance may be intrinsic or mediated by transferable carbapenemase-encoding genes [32]. This finding indicates the need for antibiotic stewardship interventions to improve the appropriate use of carbapenems and reduce the emergence of carbapenem resistance among Gram-negative bacilli. Bacteremia and pneumonia were the most common infections caused by CR-GNB pathogens, and this is in consonance with the results of previous studies conducted in China and the United States [14,29]. This could be attributed to the use of invasive devices such as intubation/mechanical ventilation, enteral and parenteral feeding tubes, hemodialysis and central as well as peripheral catheterisation. Therefore, infection control and prevention strategies should be strengthened in patients exposed to invasive devices to prevent the transmission of resistant pathogens. This can be achieved by developing a flagging system for CRE, CRAB and CRPA colonisation to identify patient at risk earlier and implement appropriate measures such as patient isolation and contact precaution to prevent cross transmission

The current study revealed that meropenem and piperacillin-tazobactam were the most commonly used antibiotics for empiric therapy. This is consistent with the result of a study conducted in China [10]. Meropenem and piperacillin-tazobactam are frequently used as empiric therapy for patients with suspected extended-spectrum beta-lactamase infection [33,34], and this explains the high rate of usage of these antibiotics in patients with infection caused by CR-GNB. In practice, clinicians prefer to use combination antibiotic therapy for treatment of CR-GNB infections [4,35]. However, there is limited evidence to support the use of combination therapy. More than one-thirds of the patients were treated with combination empiric therapy in the current study. This result, coupled with the delay in initiating effective antibiotic therapy, indicates that most cases received inappropriate empiric antibiotics. A previous study reported that only one-third of infections caused by CR-GNB received appropriate empiric antibiotic treatment [36]. Our study revealed that only 11% of the patients received appropriate empiric antibiotic treatment. Inappropriate empiric antibiotic therapy is associated with increased risk of mortality in CR-GNB infections [36]. Therefore, antimicrobial stewardship interventions are recommended to promote the early administration of effective antibiotics and improve the use of antibiotics particularly in combination with antibiotic therapy. Inappropriate combination therapy may contribute to the emergence of antimicrobial resistance and there is limited evidence to support the use of combination therapy in routine clinical practice [37]. This highlights the need for hospital pharmacists to provide antibiotic stewardship services and improve the use of antibiotics in the management of multidrug-resistant infections. The participation of hospital pharmacists in antimicrobial stewardship programs is limited [38] and future pharmacists are not well prepared to participate in stewardship activities [39]. Therefore, training practicing pharmacists and the incorporation of antimicrobial stewardship into undergraduate and postgraduate pharmacy curriculum is recommended [40].

Overall, in-hospital and 14-day mortality rate was 41.4% and 23.4%, respectively. Multivariate logistic regression analysis showed that sepsis or septic shock is independently associated with mortality and this corroborates the result of previous studies [36,41,42]. Sepsis/septic shock is a prominent predictor of mortality particularly when there is a delay in the administration of effective antibiotics [43]. CR-GNB infections are commonly associated with late diagnosis, high bacterial virulence and inappropriate antibiotic therapy; therefore, most cases will progress to sepsis/septic shock [44]. Therefore, interventions to promote the early administration of effective antibiotics and hemodynamic support within the early hours of recognition of sepsis/septic shock is recommended to reduce the risk of mortality. In addition, ICU admission is significantly associated with mortality because ICU patients are more likely to have severe underlying diseases and several comorbidities. The result also showed that patients who had elevated serum creatinine had 2.7 times higher risk of death compared to those without elevated serum creatinine. Elevated serum creatinine is an indicator of kidney injury. This could be attributed to either complications of infection (acute kidney injury in sepsis) or adverse effects from antibiotics used for the treatment of infection. Colistin and amikacin were the most commonly used definitive therapy in the current study and evidence has demonstrated a higher rate of kidney injury associated with the use of colistin in the management of CR-GNB infections [45,46]. Therefore, renal function should be closely monitored among patients with CR-GNB infections and renal dose adjustment should be administered in those with renal impairment.

Evidence has shown that inappropriate empiric antibiotic therapy increases the risk of mortality in patients with infections due to CR-GNB pathogens [47,48]. The current study revealed that inappropriate empiric antibiotics did not significantly increase mortality risk. This could be attributed to the small sample size in the current study. In addition, the mean duration before active antibiotics was 4.6 days which indicates a delay in the administration of effective treatments. These drug therapy issues are important opportunities for hospital pharmacists to improve the early administration of effective antibiotics among patients with multidrug-resistant infections. This study has several limitations, and the results should be interpreted with caution. Firstly, a retrospective study design was used and this design has some weaknesses in establishing the association between variables. Secondly, the study was conducted in a single hospital and this may limit the generalisability of the findings. Future studies should include a large sample and multiple centres. Thirdly, there are few missing data in the electronic medical records and this leads to an assessment bias in the study. Despite the limitations, this study provides an insight into the pattern of treatment and the factors that predict mortality in patients with infections caused by CR-GNB.

## 5. Conclusions

There are limited choices of antibiotics for the treatment of CR-GNB infections. Inappropriate empiric therapy including the use of combination antibiotic therapy and delays in the initiation of active antibiotics is common in the treatment of infections caused by CR-GNB pathogens. Carbapenems and beta-lactam and beta-lactamase inhibitor combinations are the most commonly used empiric antibiotics. The mortality rate among patients with infections caused by CR-GNB is high and it is independently associated with ICU admission, sepsis/septic shock and elevated serum creatinine. Antibiotic stewardship interventions aimed at promoting early administration and appropriate use of antibiotics are recommended. Future research with a larger patient population and multiple centres is recommended.

## Figures and Tables

**Table 1:** Demographic and clinical characteristics of the patients.

Variable	Frequency (%) *N* = 145
Mean age (SD) in years	57.9 ± 15.8
Gender	
Male	99 (68.3)
Female	46 (31.7)
Race	
Malay	128 (88.3)
Chinese	6 (4.1)
Indian	9 (6.2)
Others	2 (1.4)
Intensive care unit admission	84 (57.9)
Prior carbapenem use in the last 30 days	58 (40.0)
Immunocompromised patient	36 (24.8)
Presence of any comorbidity	131 (90.3)
Mean (SD) Charlson comorbidity index score	3.6 ± 2.2
Type of comorbidity	
Hypertension	89 (61.4)
Diabetes mellitus	76 (52.4)
Malignancy	28 (19.3)
Chronic kidney disease	26 (17.9)
Dyslipidemia	20 (13.8)
Ischemic heart disease	16 (11.0)
Stroke	15 (10.3)
Sepsis/septic shock	93 (64.1)
Type of infection	
Pneumonia	59 (40.7)
Bacteremia	37 (25.5)
Skin and soft tissue infection	20 (13.8)
Urinary tract infection	19 (13.1)
Intra-abdominal infection	5 (3.4)
Osteomyelitis	4 (2.8)
Ventriculitis	1 (0.7)
Acquisition type of infection	
Hospital-acquired	107 (73.8)
Community-acquired	38 (26.2)
Recent hospitalisation	78 (53.8)
Recent surgery	74 (51.0)
Patient’s parameters on the first day of infection	
Intubation	78 (53.8)
Hemodynamic (ionotrope) support	60 (41.4)
Fever	48 (33.1)
Leucocytosis	104 (71.7)
Tachycardia	68 (46.9)
Mean lowest systolic blood pressure	108 ± 22.1
Mean lowest diastolic blood pressure	61.2 ± 12.6
Elevated serum creatinine	75 (51.7)
Elevated serum urea	85 (58.6)
Low haemoglobin level	116 (80.0)
Thrombocytopenia	32 (22.1)
Hypoalbuminemia	117 (80.7)
Hyperbilirubinemia	39 (26.9)
Elevated aspartate transaminase	80 (55.2)
Elevated alanine transaminase	55 (37.9)
Elevated alkaline phosphatase	85 (58.6)

SD: standard deviation.

**Table 2:** Antibiotics used for empirical and definitive therapy among the patients.

Variable	Frequency (%)
Empirical antibiotics (*n* = 235)	
Meropenem	58 (24.7)
Piperacillin-tazobactam	48 (20.4)
Vancomycin	19 (8.1)
Colistin	13 (5.5)
Cotrimoxazole	11 (4.7)
Ciprofloxacin	11 (4.7)
Ceftriaxone	11 (4.7)
Cefuroxime	10 (4.3)
Amoxicillin-clavulanic acid	8 (3.4)
Metronidazole	8 (3.4)
Cefepime	7 (3.0)
Ampicillin-sulbactam	6 (2.6)
Ceftazidime	5 (2.1)
Azithromycin	5 (2.1)
Cloxacillin	4 (1.7)
Amikacin	3 (1.3)
Clindamycin	3 (1.3)
Imipenem	2 (0.9)
Fusidic acid	1 (0.4)
Ertapenem	1 (0.4)
Cefoperazone	1 (0.4)
Combination empirical antibiotic therapy	55 (37.9)
Received active empirical antibiotics	16 (11.0)
Received active antibiotic treatment	87 (60.0)
Received active antibiotic treatment in first 48 h	21 (14.5)
Definitive antibiotics (*n* = 109)	
Colistin	69 (63.3)
Amikacin	9 (8.3)
Meropenem	6 (5.5)
Piperacillin-tazobactam	6 (5.5)
Gentamicin	5 (4.6)
Ceftazidime	4 (3.7)
Cotrimoxazole	3 (2.8)
Ciprofloxacin	3 (2.8)
Cefepime	2 (1.8)
Imipenem	2 (1.8)
Combination definitive antibiotic therapy	62 (42.8)
Types of combinations	
Colistin-based combinations	47 (75.8)
Non-colistin-based combinations	15 (24.2)
Mean days to effective antibiotics	4.6 ± 3.3

**Table 3:** Univariate logistic regression analysis for factors associated with in-hospital mortality.

Variable	Odds Ratio	95% Confidence Interval	*p*-Value
Age	1.006	0.984–1.027	0.605
**Female gender**	**2.842**	**1.383–5.842**	**0.004**
Race			
Malay	Reference		
Chinese	0.731	0.129–4.137	0.723
Indian	1.827	0.468–7.127	0.386
Others	1.462	0.089–23.895	0.790
**Intensive care unit admission**	**10.766**	**4.537–25.544**	**<0.001**
Receive active empirical antibiotics	1.115	0.391–3.181	0.838
Immunocompromised	1.182	0.552–2.529	0.667
Charlson comorbidity index	1.106	0.950–1.287	0.195
**Sepsis/septic shock**	**5.802**	**2.539–13.257**	**<0.001**
Type of infection	1.763	0.806–3.857	0.156
Diagnosis			
Bacteremia	1.489	0.702–3.157	0.300
**Pneumonia**	**2.176**	**1.104–4.291**	**0.025**
**Urinary tract infection**	**0.227**	**0.063–0.818**	**0.023**
Skin and soft tissue infection	0.424	0.145–1.239	0.117
Intra-abdominal infection	0.943	0.153–5.820	0.949
Polymicrobial infections	1.000	0.438–2.285	1.000
Days to effective antibiotics	0.999	0.878–1.136	0.983
Highest temperature	0.891	0.585–1.348	0.585
Fever	0.868	0.427–1.765	0.696
**Leucocytosis**	**3.563**	**1.412–8.990**	**0.007**
Tachycardia	1.383	0.701–2.725	0.349
**Elevated serum creatinine**	**2.881**	**1.411–5.881**	**0.004**
**Elevated serum urea**	**3.448**	**1.611–7.377**	**0.001**
Anemia	2.613	0.891–7.662	0.080
**Thrombocytopenia**	**2.302**	**1.025–5.170**	**0.043**
**Intubation**	**10.091**	**3.580–28.444**	**<0.001**
**Hyperbilirubinemia**	**2.178**	**1.003–4.729**	**0.049**
Pathogen			
*Enterobacteriaceae*	Reference		
*P. aeruginosa*	0.752	0.278–2.033	0.574
*Acinetobacter baumannii*	1.015	0.469–2.197	0.970
**Received active antibiotic treatment**	**3.071**	**1.491–6.327**	**0.002**
Received active antibiotic treatment in first 48 h	1.261	0.486–3.271	0.633

Bold font indicates statistical significance.

**Table 4:** Multivariate logistic regression analysis for factors associated with in-hospital mortality.

Variable	Adjusted Odds Ratio	95% Confidence Interval	*p*-Value
**Intensive care unit admission**	**5.201**	**1.603–16.872**	**0.006**
**Sepsis/septic shock**	**3.430**	**1.021–11.522**	**0.049**
**Elevated serum creatinine**	**2.752**	**1.005–7.536**	**0.049**
Received active antibiotic treatment	2.562	0.896–7.329	0.079

Bold font indicates statistical significance.

## Data Availability

All data generated or analyzed during this study are included in this article. Further enquiries can be directed to the corresponding author.

## Supplementary Materials

The following are available online at https://www.mdpi.com/article/10.3390/tropicalmed7120415/s1, Table S1: Microbiological characteristics of CRGNB infections; Table S2: Antimicrobial susceptibility profile of the CRGNB isolates.
