# Supplementary material for: Treatments and Predictors of Mortality for Carbapenem-Resistant Gram-Negative Bacilli Infections in Malaysia: A Retrospective Cohort Study
Source: Trop Med Infect Dis. 2022 Dec 2;7(12):415. doi: 10.3390/tropicalmed7120415 (PMC9783639; doi:10.3390/tropicalmed7120415)
Supplement: Supplementary file 1 [file tropicalmed-07-00415-s001.zip › tropicalmed-2047580-supplementary.pdf]

Table S1: Microbiological characteristics of CRGNB infections

**Variable**	**Frequency**	**Percentage**
Pathogen		
Enterobacteriaceae	40	
Klebsiella pneumonia	**33**	**22.8**
Escherichia coli	3	2.1
Enterobacter spp	2	1.4
Serratia marcescens	2	1.4
Acinetobacter baumannii	77	53.1
Pseudomonas aeruginosa	**28**	**19.3**
Concurrent fungal infection	21	14.5
Polymicrobial infection	29	20.0
Other pathogens involved in polymicrobial infections		
Enterobacteriaceae	14	28.0
k. pneumonia	4	2.8
E. coli	2	1.4
Proteus spp	2	1.4
Citrobacter spp	4	2.8
Pseudomonas aeruginosa	3	2.1
Candida spp	4	2.8
Staphylococcus spp	5	3.5
Stenotrophomonas maltophilia	2	1.4
Acinetbacter baumannii	2	1.4
Enterococcus spp.	4	2.8
Burkholderia cepacia	1	0.7
Rhizobium radiobacter	1	0.7
Elizabethkingia meningoseptica	1	0.7

Bold front indicates statistical significant.

Table S2: Antimicrobial susceptibility profile of the CRGNB isolates

Antibiotics	Overall			CRE			CRPA			CRAB			P value
	Resistant	Intermed.	Sensitive	Resistant	Intermed.	Sensitive	Resistant	Intermed.	Sensitive	Resistant	Intermed.	Sensitive	
	n (%)	n (%)	n (%)	n (%)	n (%)	n (%)	n (%)	n (%)	n (%)	n (%)	n (%)	n (%)	
Ampicillin (63)	63 (100)	0 (0.0)	0 (0.0)	38 (100)	0 (0.0)	0 (0.0)	-	-	-	25 (100)	0 (0.0)	0 (0.0)	1.000
Amoxicillin (9)	9 (100)	0 (0.0)	0 (0.0)	4 (100)	0 (0.0)	0 (0.0)	4 (100)	0/4 (0.0)	0/4 (0.0)	1 (100)	0 (0.0)	0 (0.0)	1.000
Amoxicillin-clavulanic acid (56)	56 (100)	0 (0.0)	0 (0.0)	38 (100)	0 (0.0)	0 (0.0)	-	-	-	18 (100)	0 (0.0)	0 (0.0)	1.000
Ampicillin-sulbactam (17)	17 (100)	0 (0.0)	0 (0.0)	2 (100)	0 (0.0)	0 (0.0)	-	-	-	15 (100)	0 (0.0)	0 (0.0)	1.000
Piperacillin-tazobactam (135)	126 (93.3)	7 (5.2)	2 (1.5)	35 (94.6)	1 (2.7)	**1 (2.7)**	16 (72.7)	5 (22.7)	**1 (4.5)**	75 (98.7)	1 (1.3)	**0 (0.0)**	< **0.001***
Cefuroxime (57)	57 (100)	0 (0.0)	0 (0.0)	37 (100)	0 (0.0)	0 (0.0)	-	-	-	20 (100)	0 (0.0)	0 (0.0)	1.000
Cefotaxime (76)	66 (95.7)	3 (4.3)	0 (0.0)	34 (97.1)	1 (2.9)	0 (0.0)	3 (100)	0 (0.0)	0 (0.0)	29 (93.5)	2 (6.5)	0 (0.0)	0.648*
Ceftriaxone (14)	12 (85.7)	2 (14.3)	0 (0.0)	6 (100)	0 (0.0)	0 (0.0)	-	-	-	6 (75.0)	2 (25.0)	0 (0.0)	0.473*
Ceftazidime (142)	129 (89.0)	4 (2.8)	9 (6.2)	35 (94.6)	1 (2.7)	**1 (2.7)**	21 (75.0)	1 (3.6)	**6 (21.4)**	73 (94.8)	2 (2.6)	**2 (2.6)**	**0.012***
Cefepime (95)	86 (90.5)	2 (2.1)	7 (7.4)	34 (91.9)	0 (0.0)	**3 (7.5)**	20 (76.9)	2 (7.7)	**4 (15.4)**	32 (100.0)	0 (0.0)	**0 (0.0)**	**0.014***
Ciprofloxacin (136)	122 (89.7)	6 (4.1)	8 (5.5)	28 (82.4)	4 (11.8)	**2 (5.9)**	23 (85.2)	2 (7.4)	**2 (7.4)**	71 (94.7)	0 (0.0)	**4 (5.4)**	**0.025***
Cotrimoxazole (68)	56 (82.4)	0 (0.0)	12 (17.6)	29 (90.6)	0 (0.0)	**3 (7.5)**	8 (100)	0 (0.0)	**0 (0.0)**	19 (67.9)	0 (0.0)	**9 (32.1)**	**0.042***
Nitrofurantoin (6)	4 (66.7)	2 (33.3)	0 (0.0)	4 (80)	1 (20.0)	0 (0.0)	0 (0.0)	1 (100.0)	0 (0.0)	-	-	-	0.333*
Gentamicin (131)	94 (71.8)	6 (4.6)	31 (23.7)	16 (53.3)	2 (6.7)	**12 (40.0)**	12 (46.2)	3 (11.5)	**11 (42.3)**	66 (88.0)	1 (1.3)	**8 (10.4)**	< **0.001***
Amikacin (103)	68 (66.0)	8 (5.5)	27 (26.2)	7 (35.0)	2 (10.0)	**11 (55.0)**	8 (38.1)	4 (14.3)	**9 (42.9)**	53 (85.5)	2 (3.2)	**7 (11.3)**	< **0.001***
Ertapenem (82)	54 (85.7)	6 (9.5)	3 (4.8)	26 (74.3)	6 (17.1)	3 (8.6)	4 (100)	0 (0.0)	0 (0.0)	24 (100)	0 (0.0)	0 (0.0)	0.070*
Imipenem (143)	132 (92.3)	4 (2.8)	7 (4.9)	27 (71.1)	4 (10.5)	**7 (18.4)**	-	-	-	-	-	-	< **0.001***
Meropenem (142)	130 (89.7)	4 (2.8)	8 (5.6)	27 (71.1)	3 (7.9)	**8 (20.1)**	-	-	-	75 (98.7)	1 (1.3)	0 (0.0)	< **0.001***
Colistin (28)	0 (0.0)	0 (0.0)	28 (100)	0 (0.0)	0 (0.0)	9 (100.0)	0 (0.0)	**0 (0.0)**	**5 (100.0)**	0 (0.0)	0 (0.0)	14 (100.0)	1.000

Bold front indicates statistical significant, * p<0.05.
